# Application of cysteinyl prolyl ester for the synthesis of cyclic peptides containing an RGD sequence and their biological activity measurement

**DOI:** 10.3389/fchem.2024.1391678

**Published:** 2024-05-30

**Authors:** Akina Yamada, Toshiki Takei, Toru Kawakami, Yukimasa Taniguchi, Kiyotoshi Sekiguchi, Hironobu Hojo

**Affiliations:** Institute for Protein Research, Osaka University, Suita, Osaka, Japan

**Keywords:** cyclic peptide, cysteinyl prolyl ester, native chemical ligation, RGD, inhibitor

## Abstract

Cysteinyl RGD-peptidyl cysteinyl prolyl esters, which have different configurations at the cysteine and proline residues, were synthesized by the solid-phase method and cyclized by the native chemical ligation reaction. Cyclization efficiently proceeded to give cyclic peptides, regardless of the difference in the configuration. The peptides were further derivatized to the corresponding desulfurized or methylated cyclic peptides at the Cys residues. The inhibition activity to αvβ6 integrin binding was then analyzed by ELISA. The results showed that the activity varied depending on the difference in the configuration and modification of the cysteinyl prolyl ester (CPC) moiety, demonstrating the usefulness of this method in the search for a good inhibitor of the protein–protein interaction.

## 1 Introduction

**FIGURE 1:**
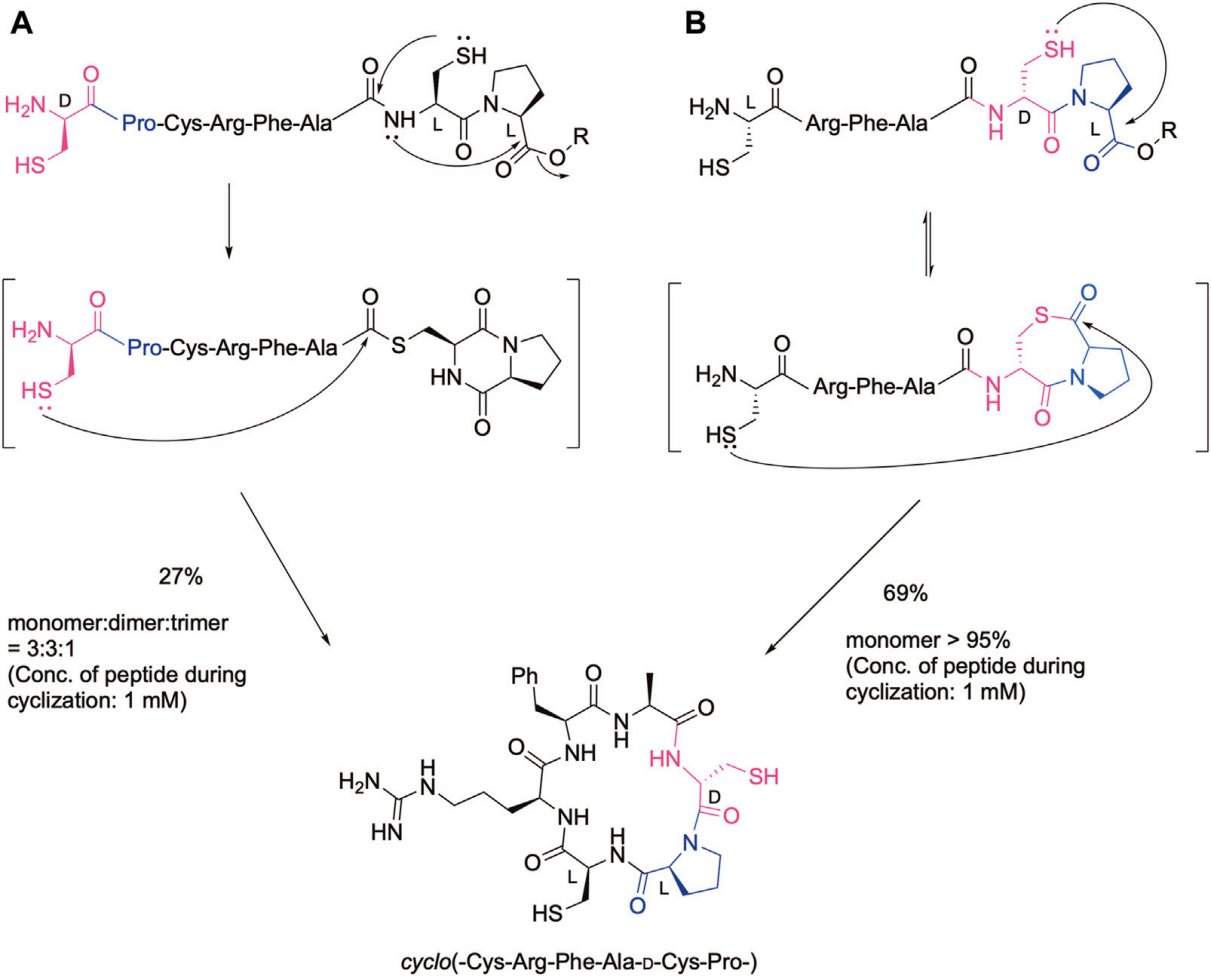
Cyclic peptide synthesis using the CPE method. **(A)** In the case of the configuration of the cysteinyl proline residue is the same, **(B)** In the case of the configuration of the cysteinyl proline residue is different.

Cyclic peptides are promising leads for drugs regarding their stability to proteinases as well as the rigidity of the cyclic structure. In fact, many cyclic peptides are currently used as therapeutics (Muttenthaler et al., 2021; Ji et al., 2023). Therefore, efficient methods have been developed to obtain cyclic peptides via the amide bond as well as the disulfide bond, oxime bond, and other bond formations (Malesevic et al., 2004; Tulla-Puche and Barany, 2004; White and Yudin, 2011; Hemu et al., 2013; Terrier et al., 2017; Gless and Olsen, 2018; Shimodaira et al., 2018; Arbour et al., 2019; Chow et al., 2019; Reguera and Rivera, 2019; Sarojini et al., 2019; Serra et al., 2020). We also developed efficient cyclization methods based on our cysteinyl prolyl ester (CPE) (Kawakami et al., 2022), which was originally developed for the synthesis of the peptide thioester (Kawakami and Aimoto, 2007). In the CPE method, a peptide with the cysteinyl prolyl ester at the C-terminus is converted to the peptide thioester via the N-to-S acyl shift, followed by diketopiperazine formation. Therefore, if a peptide has the N-terminal Cys and C-terminal CPE, in which the configuration of the cysteinyl proline residue in CPE is the same, the cyclic peptide was obtained by the native chemical ligation reaction (Dawson et al., 1994) as shown in Figure 1A. In this case, the Cys-Pro sequence was liberated from the sequence. The yield of the desired cyclic monomer was moderate due to the formation of a cyclic dimer and trimer. In contrast, when the configuration in the Cys-Pro sequence of CPE is different (LD or DL), the ligation with the N-terminal cysteine efficiently occurs at the Pro, keeping the CPC sequence via thiolactone formation (Figure 1B). Remarkably, this procedure gives the desired cyclic peptide in an excellent isolated yield without the production of cyclic dimers and oligomers, showing the efficiency of this method for cyclic peptide synthesis. In addition, we can design a cyclic peptide library with different absolute configurations at the CPC moiety, leading to different biological activities. Two cysteine residues can be further modified, such as by desulfurization and alkylation, which can further increase the number of libraries. To demonstrate these possibilities, the method was used for the synthesis of cyclic hexapeptides containing the RGD sequence, and the effect of the configuration differences on their biological activities was analyzed by the integrin-binding assay.

Integrins are heterodimeric transmembrane receptors and engage in cell adhesion. Integrins are composed of α and β subunits (Hynes, 2002; Kechagia et al., 2019). So far, 18 kinds of α-subunits and 8 kinds of β-subunits have been identified, and from these subunits, 24 kinds of integrin species are formed. Integrins can interact with many ligands, including extracellular matrix (ECM) proteins. Many of the integrins recognize short peptide sequences within the ligands. Depending on the type of recognition motif, integrins are classified into five types: the RGD-binding integrin, laminin-binding integrin, collagen-binding integrin, leukocyte-binding integrin, and EMILIN-binding integrin. As the aberrant integrin-ligand interaction is known to be involved in various diseases, the integrin inhibitor attracts much attention as a therapeutic.

**FIGURE 2:**
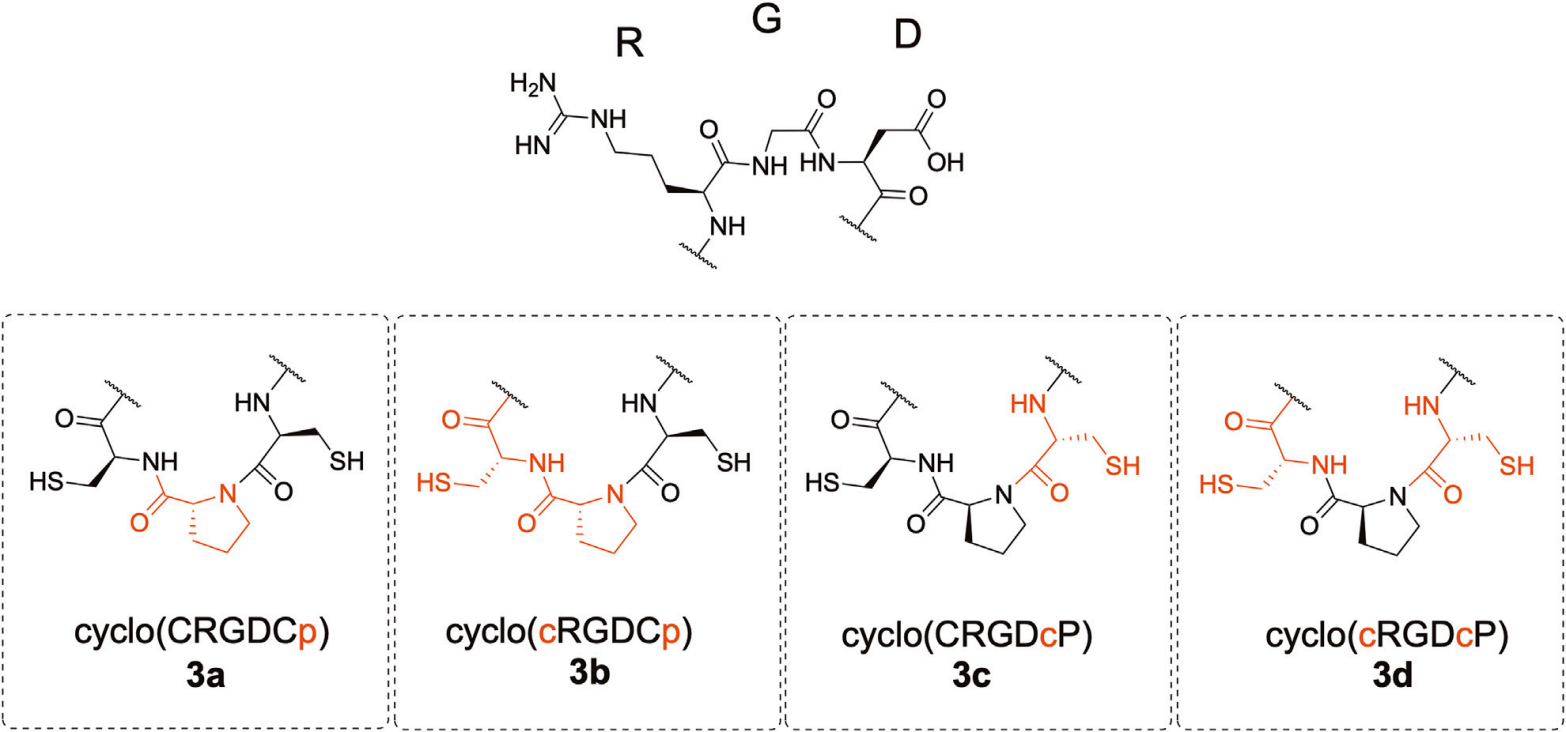
Structure of the cyclic peptides.

We synthesized cyclic hexapeptides with the Arg-Gly-Asp (RGD) sequence containing CpC, Cpc, cPC, and cPc (lowercase letters denote D-amino acids) (Figure 2). The desulfurized and methylated derivatives of these peptides were also prepared, and their inhibitory activity regarding the integrin-ligand interaction was analyzed.

## 2 Results and discussion

### 2.1 Synthesis of the linear peptides containing the Arg-Gly-Asp sequence

**FIGURE 3:**
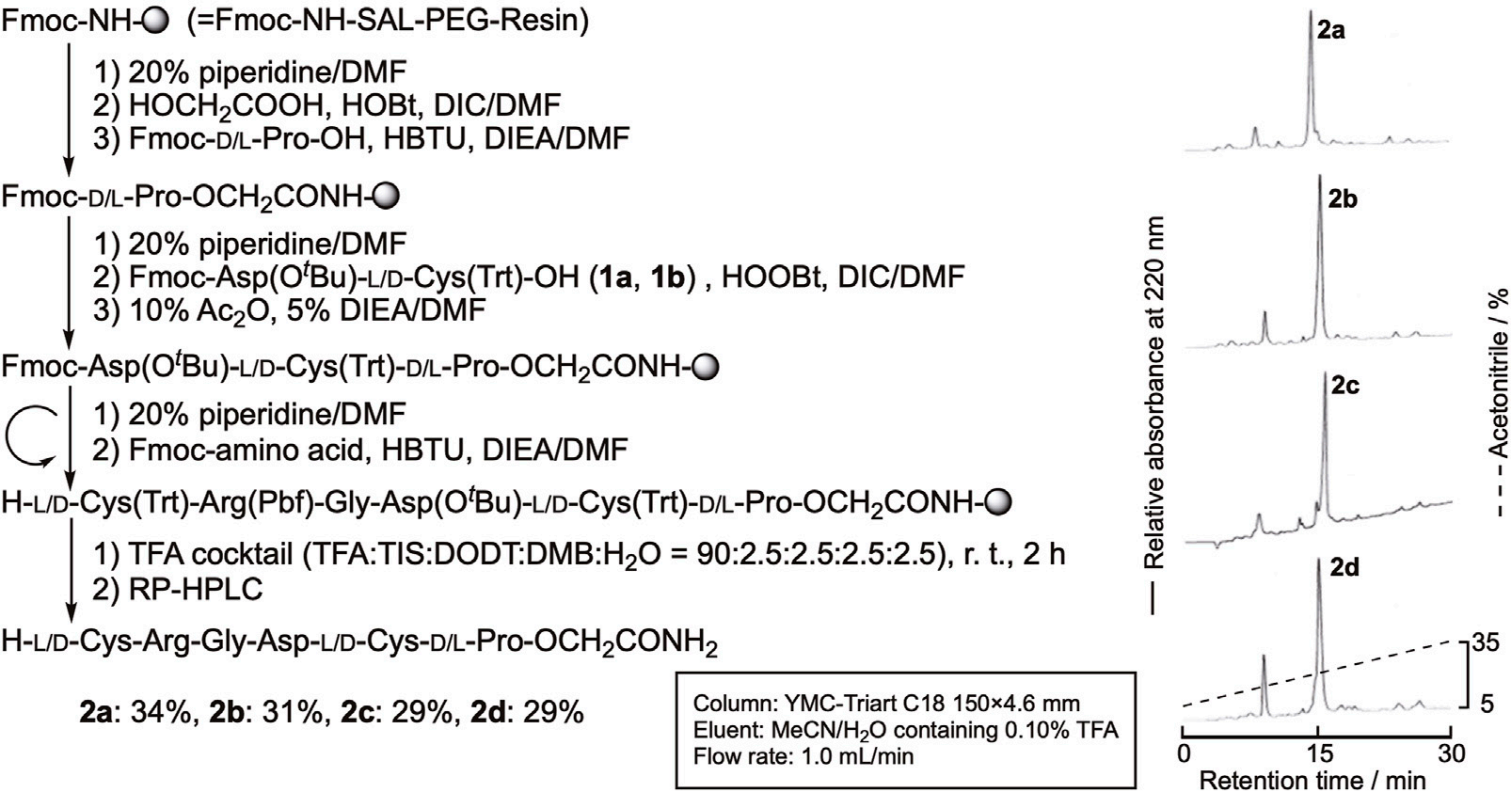
Synthetic route of the linear precursor and the RP-HPLC profile of crude peptides.

We first synthesized linear precursors, the peptide prolyl esters with different configurations, using the fluorenylmethoxycarbonyl protecting group (Fmoc) method, as shown in Figure 3. Glycolic acid was introduced to the Fmoc-NH-SAL-PEG resin using the N,N'-diisopropylcarbodiimide-1-hydroxybenzotriazole (DIC-HOBt) method. Fmoc-Pro-OH or Fmoc-D-Pro-OH was then introduced by 1-[bis(dimethylamino)methylene]-1H-benzotriazolium 3-oxide hexafluorophosphate (HBTU/DIEA). The next two amino acid residues were introduced as a dipeptide to avoid the elimination of the C-terminal dipeptide by diketopiperazine formation (Kawakami and Aimoto, 2007). The remaining amino acids were introduced using the Fmoc method to obtain the protected peptide resins with the desired sequences. The resins were then treated with the trifluoroacetic acid (TFA) cocktail to deprotect the peptides. The obtained crude peptide was purified by reversed-phase (RP)-HPLC to obtain the desired linear peptides, **2a**−**2d**, in high purity (Supplementary Figure SI-1). The yields of the peptides were around 30%.

### 2.2 Cyclization reaction

**FIGURE 4:**
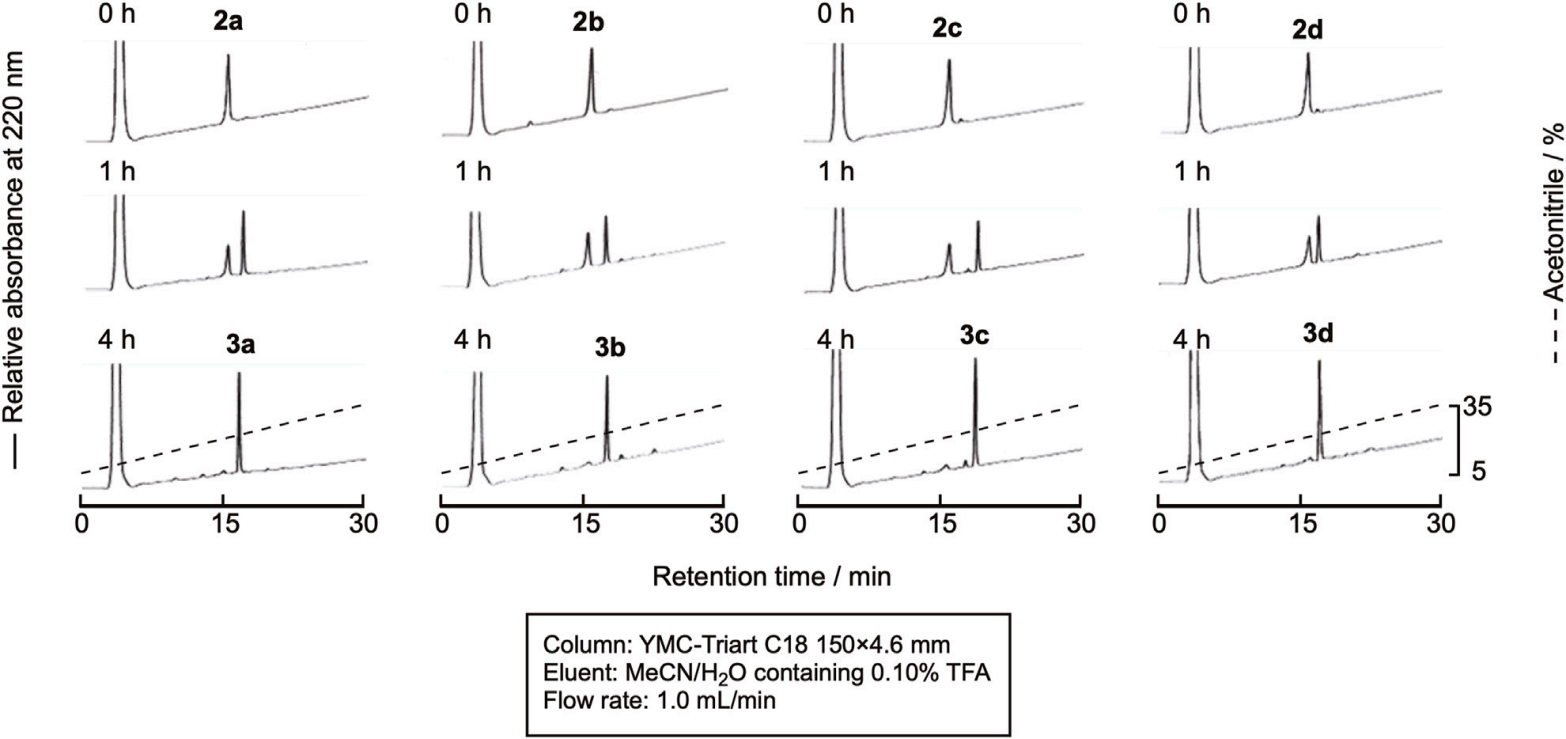
RP-HPLC profile of the cyclization reaction progress.

The linear peptide was then cyclized under the native chemical ligation reaction condition using the CPE method. In brief, the linear precursor was dissolved in a sodium phosphate buffer containing 20 mM TCEP and 20 mM ascorbic acid (pH 7.8) at the concentration of 2 mM, and the reaction mixture was kept at 37°C. In spite of the relatively high concentration of peptides, intramolecular cyclization proceeded without the formation of a dimer and oligomers within 4 h, as shown in Figure 4, indicating that the peptide cysteinyl prolyl ester tends to assume a cyclizable conformation, at least in the case of the hexapeptide. The cyclized peptides, **3a**-**3d**, were isolated by RP-HPLC, as shown in Supplementary Figure SI-2, in yields of around 50%, proving the efficient cyclization of the linear peptides by using this method.

### 2.3 Desulfurization

a) Use of VA044 as a radical initiator

**FIGURE 5:**
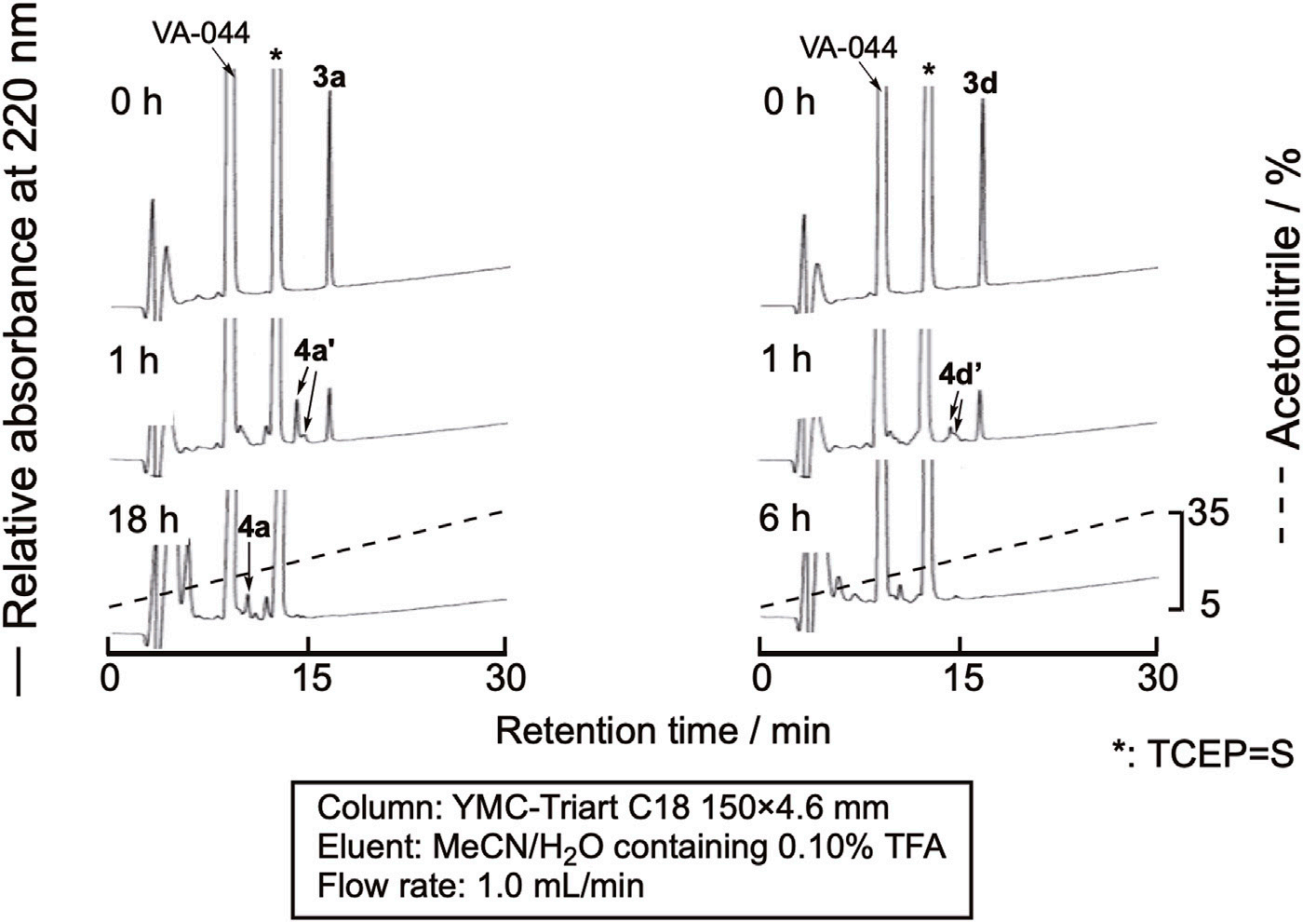
RP-HPLC profile of the desulfurization reaction using VA-044 as a radical initiator. 4a’ and 4d’ denote the peaks of peptides in which one of the cysteine residues in the sequence was desulfurized.

The desulfurization reaction was first attempted by the prevalent conditions using VA-044 as the initiator (Wan and Danishefsky, 2007). The cyclic peptides were dissolved in degassed sodium phosphate buffer (pH 6.8) containing 10 mM VA-044, 0.10 M TCEP・HCl, and 50 mM MESNa (pH 6.8) at a 1.0 mM concentration and shaken at 37 °C. Excess amounts of VA044, MESNa, and TCEP are required for this method. Unfortunately, the large peaks derived from these reagents were eluted close to the peptides, which made monitoring the progress of the reaction by RP-HPLC difficult (Figure 5). The TCEP-sulfide (TCEP = S) formed by the reaction of TCEP with MESNa eluted very close to the peptides. Therefore, we examined the use of another desulfurization reaction.

b) Use of NaBEt_4_ as the radical initiator

**FIGURE 6:**
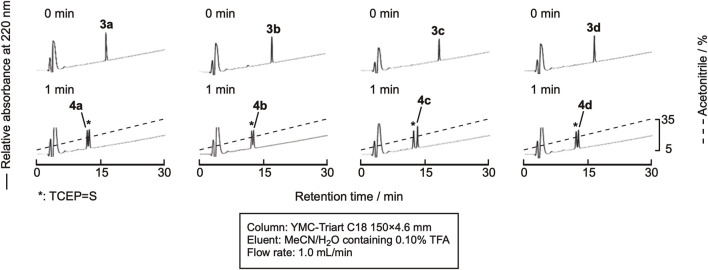
RP-HPLC profile of the desulfurization reaction.

Sun et al. recently reported the use of NaBEt_4_ as the radical initiator for desulfurization (Sun et al., 2022). As TCEP = S is derived only from the reaction of TCEP and cysteine residues, the amount is low, and monitoring the reaction by RP-HPLC would be much easier. The peptide was dissolved in 0.10 M citric acid containing 0.10 M TCEP•HCl (pH 5.2), and NaBEt_4_ was added to the mixture to the final concentration of 0.10 M in the solution. As expected, the efficient monitoring of the reaction was achieved, as shown in Figure 6. After 1 min, the reaction was complete. The mixture was purified by RP-HPLC to obtain the desulfurized peptides, **4a**-**4d** (Supplementary Figure SI-3). The isolated yield of the product was around 70%, indicating a clean reaction.

### 2.4 Methylation

**FIGURE 7:**
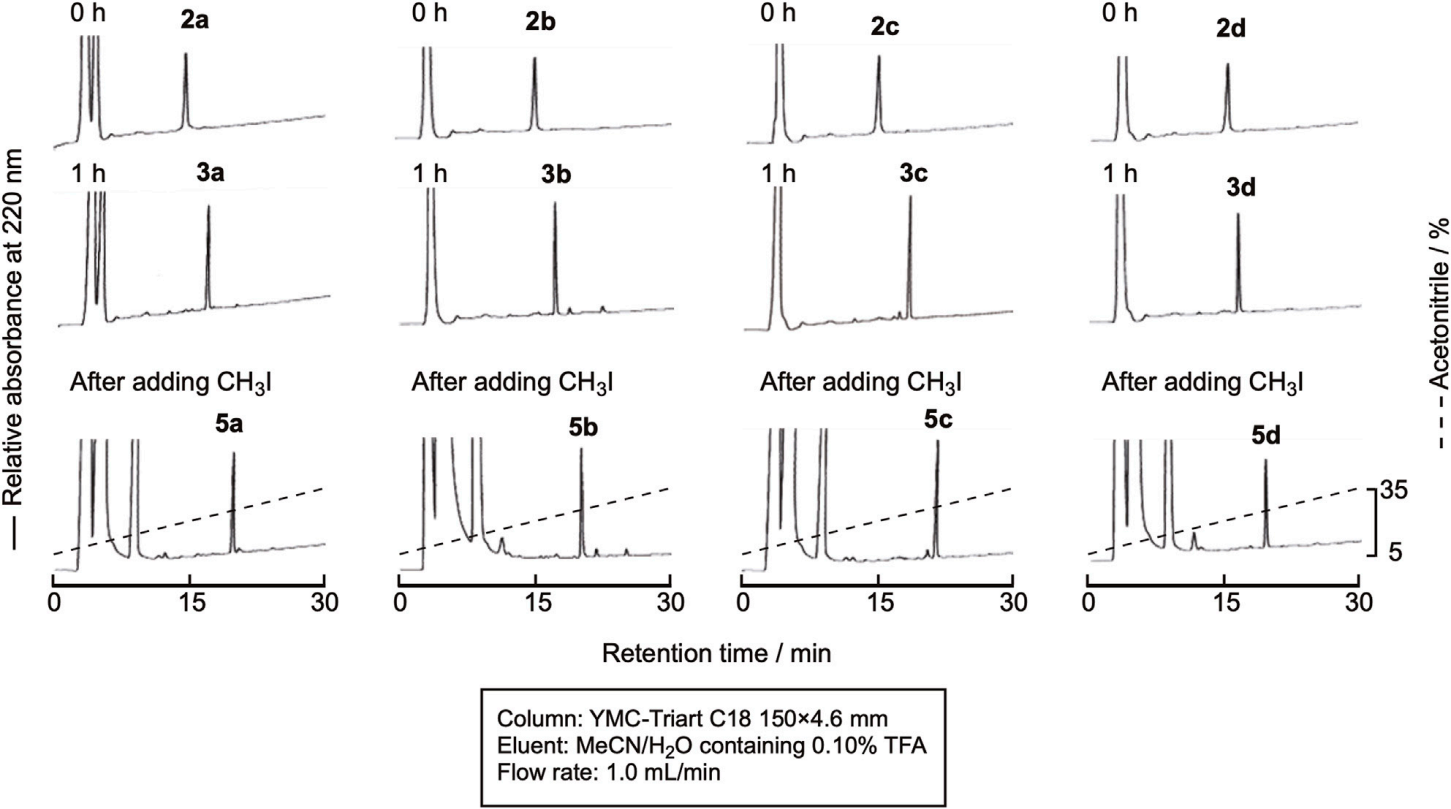
RP-HPLC profile of the cyclization and subsequent methylation reaction.

In this experiment, the cyclization and methylation were sequentially performed in one pot. The cyclization of linear peptides was carried out in the same manner as described in Section 2.2 Cyclization, and the reaction proceeded similarly. Without isolating the product, CH_3_I in dimethylformamide (DMF) was added, and the reaction mixture was shaken for 30 min at room temperature. The reaction efficiently proceeded, and the doubly methylated peptide was obtained, as shown in Figure 7. Isolation by RP-HPLC gave the methylated peptides **5a**-**5d** in high purity (Supplementary Figure SI-4) in yields of around 45%.

### 2.5 Inhibition of integrin-ligand binding by the cyclic peptides

Among the RGD-binding integrins, we selected α_v_β_6_ integrin for measurement of the inhibition activity of the cyclic peptides. The α_v_β_6_ integrin is reported to recognize the sequence beyond the RGD motif, RGDLXXL (Dong et al., 2014). Therefore, the inhibition activity of the cyclic peptides is expected to vary depending on the configuration and the modification at the CPC moiety. In addition, the inhibitor of the α_v_β_6_ integrin is still under development (John et al., 2020), while an efficient inhibitor, cilengitide, exists for the α_v_β_3_ and α_v_β_5_ integrins (Stupp and Ruegg, 2007). The latency-associated peptide (LAP, TGF-β1) was used as a ligand for α_v_β_6_ integrin and coated on the ELISA plate, to which the α_v_β_6_ integrin and cyclic peptides were added to measure the inhibitory activity of the cyclic peptides by ELISA. The inhibitory activity of the commonly used RGD tripeptide was also measured as a control.

**FIGURE 8:**
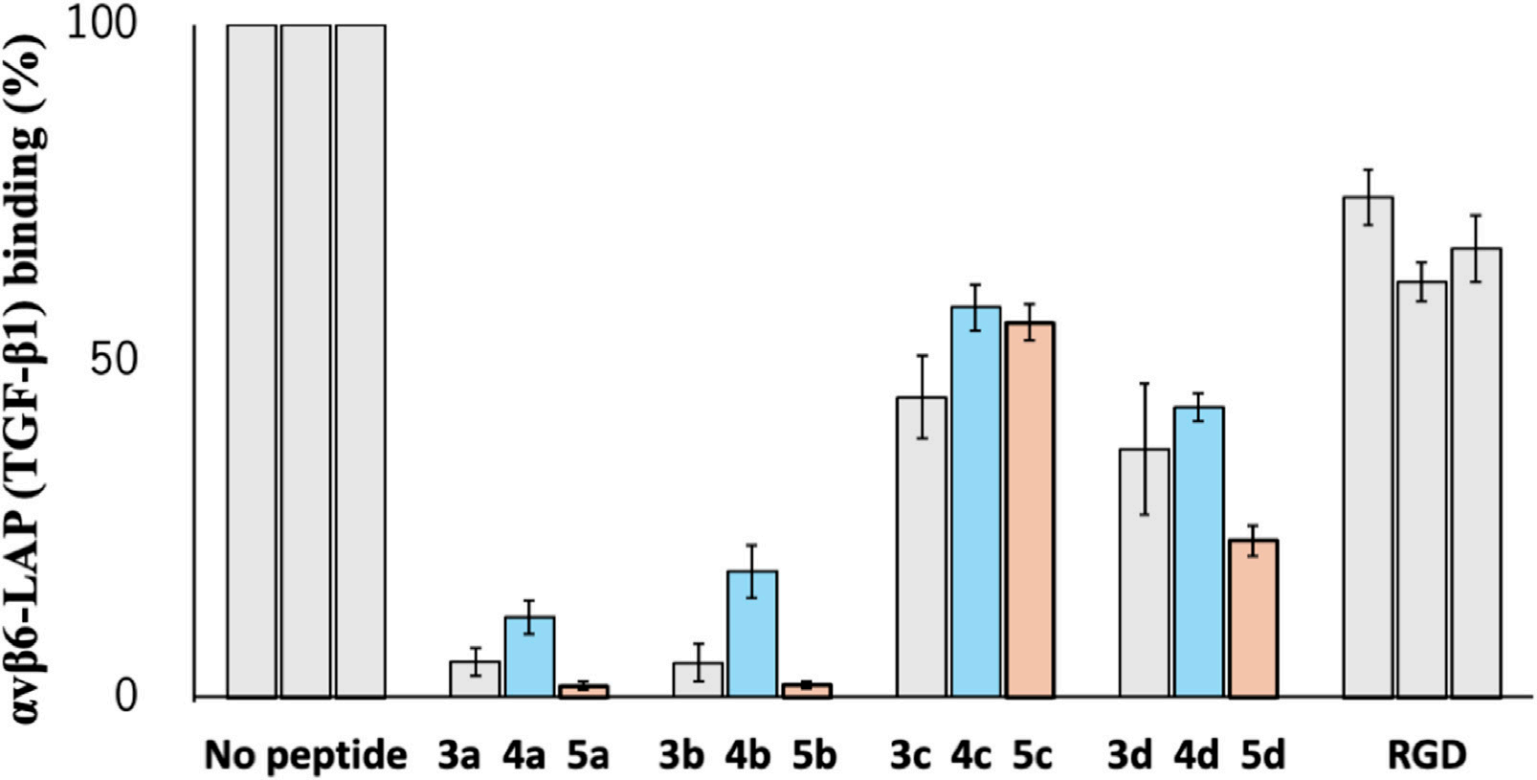
Inhibitory activity of cyclic peptides for binding of the α_v_β_6_ integrin with LAP (TGF-β1) under the following conditions: 1 µM peptide concentration, 10 nM integrin αvβ6 in the presence of 1.0 mM Mn^2+^, and LAP (TGF-β1) 10 nM.

As shown in Figure 8, the parental cyclic peptides, **3a** to **3d**, retained stronger or comparable inhibitory activity than the RGD control peptide, indicating that the RGD sequence in these cyclic peptides is recognized by α_v_β_6_ integrin. Peptides **3a** and **3b** are stronger than **3c** and **3d**, showing that the L-configuration is favored for the penultimate residue to Asp. In addition, the residue also requires hydrophobicity as the inhibitory activity of the desulfurized peptides **4a** to **4d** decreased compared to the parental peptides **3a** to **3d**. This fact is also supported by the increased inhibitory activity of the methylated peptides **5a** to **5d**, which are more hydrophobic than the parental peptides. These results agree with the fact that the α_v_β_6_ integrin recognizes the sequence RGDLXXL, where the penultimate amino acid to Asp is Leu (Dong et al., 2014). Therefore, there is a possibility that modification at the cysteine residues by more bulky hydrophobic groups will further increase the inhibitory activity, demonstrating the utility of the CPC method for the efficient peptide design.

## 3 Conclusion

The cyclic peptide synthesis using the CPE method has been established using the RGD integrin-binding motif as a model. The linear precursor was easily synthesized by the Fmoc solid-phase method and cyclized by the native chemical ligation reaction at a relatively high 2 mM concentration without the formation of dimeric and oligomeric cyclic peptides. In addition, the desulfurization and alkylation of the parental peptides at the Cys residues also efficiently proceeded, which led to an increase in the number of cyclic peptides with different conformations. The inhibition of the α_v_β_6_ integrin-binding activity measurement showed that the activity depends on the configuration of the CPC motif as well as the desulfurization and methylation at this motif, demonstrating the usefulness of this method for the design of cyclic peptide inhibitors.

## 4 Experimental procedure

### 4.1 General procedure

The amino acid derivatives used were of the L-configuration and purchased from Peptide Institute (Osaka) unless otherwise noted. H-L and D-Cys(Trt)-OH, Fmoc-NH-SAL-PEG resin (NH-SAL: Rink amide), and HOObt were purchased from Watanabe Chemical Industry (Hiroshima). The other reagents, glycolic acid, piperidine, DIEA, acetic anhydride, DMF (Nacalai Tesque, Kyoto), HOSu, HOBt, WSCI・HCl, and HBTU (Peptide Institute, Minoh), DIC (Fujifilm Wako Pure Chemicals, Tokyo) were also commercially available. Analytical thin-layer chromatography (TLC) was performed using TLC Silica gel 60 F_254_. (Merck, Darmstadt). Unless otherwise noted, materials purchased from distributors were used without any purification. The NMR spectra were recorded using an AV400 spectrometer at 400 MHz (^1^H NMR) (Bruker Corporation, MA). The chemical shifts are expressed in ppm downfield from the internal tetramethylsilane signal for the solution in the deuterated solvents. Multiplicities are given as “s” (singlet), “d” (doublet), “t” (triplet), and “m” (multiplet). Broad peaks in the NMR spectra are indicated as “br.” RP-HPLC was carried out on YMC-Triart C18 (4.6 × 150 mm) (YMC CO., LTD., Kyoto) (analytical) or YMC-ProC18 (10 × 250 mm) (YMC CO., LTD., Kyoto) (preparative) using a linearly increasing gradient of MeCN in 0.1% TFA/H_2_O. Detection was done by an absorbance measurement at 220 nm. ESI-MS was measured using LCQ DECA XP Plus (Thermo Fisher Scientific, MA) or LCQ Fleet (Thermo Fisher Scientific, MA). The amino acid composition of a peptide was determined using a LaChrom amino acid analyzer (Hitachi, Tokyo) after hydrolysis with 6 M HCl at 180°C for 30 min in an evacuated sealed tube.

The Fmoc group quantification was performed using a dried resin sample (200–500 μg), which was treated with 20% piperidine/DMF (50 µL) for 30 min. The mixture was diluted with DMF (5.0 mL), and the absorbance at 301 nm was measured by a V-730BIO (JASCO, Tokyo). The content of the Fmoc group (*x* mmol) in the 1 g of resin was calculated by the following equation:

$$x=\frac{1000\times b\times c}{\varepsilon \times a}$$

where *a* is the weight of the resin in mg, *b* is the volume of the diluted solvent in mL, *c* is the absorbance at 301 nm, and ε is the molar extinction coefficient ( ε = 7,800 (mol L^−1^) ^−1^ cm^−1^).

The cDNA encoding the extracellular region of the human integrin β6 (Met1-Ile708) was amplified using the total RNA extracted from the human colon adenocarcinoma cell line WiDr as a template. The cDNA was digested with BamHI and PmeI and ligated into the corresponding restriction sites of the pEF-BASE-His6 expression vector (REF: PMID11323715). An expression vector for the extracellular region of the human integrin av with an ACID α-helical coiled-coil peptide was kindly gifted from Dr. Junichi Takagi (Institute for Protein Research, Osaka University) (REF: PMID 12230977). A FLAG tag was fused to the C-terminal site of the integrin αv-ACID sequence as previously described (REF: PMID 16324831). An expression vector for the human LAP (TGFβ1) was constructed as previously described (REF: PMID 27033701). Recombinant human αvβ6 and LAP were expressed using the FreeStyle™ 293 Expression System and purified from the resultant supernatant, as previously shown (REF: PMID 16324831 & 27,033,701). A rabbit pAb against the ACID/BASE coiled-coil region was produced as previously described (REF: PMID 16413178). The pAb was biotinylated using EZ-Link™ Sulfo-NHS-LC-Biotin (Thermo Fisher Scientific) according to the manufacturer’s instructions.

### 4.2 Fmoc-Asp(O^*t*^Bu)-Cys(Trt)-OH (1a)

Fmoc-Asp(O^*t*^Bu)-OH (410 mg, 1.0 mmol) and HOSu (130 mg, 1.1 mmol) were dissolved in DCM (5.0 mL); then, the mixture was stirred for 15 min at 0°C. WSCD・HCl (210 mg, 1.1 mmol) was added, and the solution was stirred overnight at room temperature. H-Cys(Trt)-OH (440 mg, 1.2 mmol) and DIEA (0.40 mL, 2.3 mmol) were added, then the mixture was stirred for 2 h at room temperature. After the solution was evaporated *in vacuo*, the residue was dissolved in AcOEt, washed with 5.0% aq KHSO_4_ and brine, then dried over Na_2_SO_4_. After filtration, the solvent was removed *in vacuo*, and the residue was purified by silica gel column chromatography using hexane:AcOEt (1:2 containing 0.5% AcOH) to obtain compound **1a** (520 mg, 68%). ^1^H NMR (400 MHz, CDCl_3_) δ (ppm): 7.75 (d, *J* = 7.6 Hz, 2H, Ph-H Fmoc), 7.55 (m, 2H, Ph-H Fmoc), 7.40–7.38 (m, 8H, Ph-H Fmoc, Ph-H Trt), 7.30–7.22 (m, 8H, Ph-H Trt), 7.19–7.15 (tt, *J* = 7.2 Hz, 1.2 Hz, 3H, Ph-H Trt), 7.08 (d, *J* = 7.2 Hz, 1H, NH Cys), 5.96 (d, *J* = 7.8 Hz, 1H, NH Asp), 4.55 (dd, *J* = 11.2 Hz, 6.8 Hz, 1H, CH_α_ Asp), 4.49–4.30 (m, 2H, CH_2_ Fmoc), 4.27–4.22 (dd, *J* = 12.0 Hz, 6.8 Hz, 1H, CH_α_ Cys), 4.18 (m, 1H, CH Fmoc), 2.81 (dd, *J* = 17.2 Hz, 2.4 Hz, 1H, CH_2β_ Asp), 2.73 (dd, *J* = 12.8 Hz, 7.2 Hz, 1H, CH_2β_ Cys), 2.65 (dd, *J* = 12.8 Hz, 4.8 Hz, 1H, CH_2β_ Cys), 2.60 (dd, *J* = 17.2 Hz, 7.2 Hz, 1H, CH_2β_ Asp), and 1.41 (s, 9H, ^*t*^Bu). ESI mass, found *m*/*z* 779.251, calcd for (M + Na)^+^: 779.276.

### 4.3 Fmoc-Asp(O^*t*^Bu)-D-Cys(Trt)-OH (1b)

Starting from Fmoc-Asp(O^*t*^Bu)-OH (620 mg, 1.5 mmol), the reaction was performed following the same procedure as described for compound **1a** to obtain the titled compound **1b** (870 mg, 76%). ^1^H NMR (400 MHz, CDCl_3_) δ (ppm): 7.74 (d, *J* = 7.6 Hz, 2H, Ph-H Fmoc), 7.54 (m, 2H, Ph-H Fmoc), 7.39–7.34 (m, 8H, Ph-H Fmoc, Ph-H Trt), 7.30–7.21 (m, 8H, Ph-H Trt), 7.18–7.14 (m, 3H, Ph-H Trt), 7.11 (d, *J* = 6.8 Hz, 1H, NH Cys), 6.09 (d, *J* = 8.8 Hz, 1H, NH Asp), 4.62 (dd, *J* = 13.6 Hz, 6.8 Hz, 1H, CH_α_ Asp), 4.40–4.29 (m, 2H, CH_2_ Fmoc), 4.26–4.21 (m, 1H, CH_α_ Cys), 4.12 (dd, *J* = 14.4 Hz, 7.2 Hz, 1H, CH Fmoc), 2.82–2.64 (m, 1H, CH_2β_), and 1.40 (s, 9H, ^*t*^Bu). ESI mass, found *m*/*z* 779.246, calcd for (M + Na)^+^: 779.276.

### 4.4 H-Cys-Arg-Gly-Asp-Cys-D-Pro-OCH_2_CONH_2_ (2a)

Fmoc-NH-SAL-PEG resin (0.21 mmol/g, 710 mg, 0.15 mmol) was treated with 20% piperidine/DMF (2.0 mL) for 5 min, followed by 5 min and 10 min treatments with the fresh piperidine/DMF reagents. After washing with DMF(x5), glycolic acid (24 mg, 0.31 mmol), HOBt (47 mg, 0.35 mmol), and DIC (53 μL, 0.34 mmol) in DMF (2.0 mL) were added to the resin and vortexed overnight at room temperature. The resin was filtered and washed with DMF(x5). Fmoc-D-Pro-OH (160 mg, 0.46 mmol) activated by 0.45 M HBTU/DMF (1.0 mL, 0.46 mmol) and DIEA (120 μL, 0.69 mmol) were added, and the mixture was vortexed for 6 h at room temperature. After washing with DMF (x3) and MeOH, the resin was dried *in vacuo* to obtain Fmoc-D-Pro-OCH_2_CONH-SAL-PEG resin (760 mg, 100%). The Fmoc group on the resin was quantified as 0.21 mmol/g resin. The Fmoc group on the resin (0.15 mmol) was removed by 20% piperidine/DMF, and the resin was washed with DMF(x5). Compound **1a** (240 mg, 0.32 mmol), HOOBt (57 mg, 0.35 mmol) in DMF (2.0 mL), activated by DIC (58 μL, 0.38 mmol), was added to the resin, and the mixture was vortexed overnight at room temperature. After washing and drying, Fmoc-Asp(O^*t*^Bu)-Cys(Trt)-D-Pro-OCH_2_CO-NH-SAL-PEG resin (770 mg) was obtained. The Fmoc group on the resin was quantified as 0.15 mmol/g (78%). The obtained resin (0.12 mmol) was treated with 10% acetic anhydride-5.0% DIEA/DMF (2.0 mL), vortexed for 5 min, and then washed with DMF(x3). After the Fmoc group was removed by the same procedure, Fmoc-Gly-OH (140 mg, 0.47 mmol), activated by 0.45 M HBTU/DMF (1.0 mL, 0.45 mmol) and DIEA (160 μL, 0.93 mmol), was added to the resin, and the mixture was vortexed for1 h at room temperature. Fmoc-Arg(Pbf)-OH was introduced in the same manner, and the obtained resin, Fmoc-Arg(Pbf)-Gly-Asp(O^*t*^Bu)-Cys(Trt)-D-Pro-OCH_2_CO-NH-SAL-PEG resin, was divided into two portions. Fmoc-Cys(Trt)-OH (140 mg, 0.23 mmol) was introduced to the half of the resin (63 μmol) in the same manner, followed by the Fmoc group removal to obtain H-Cys(Trt)-Arg(Pbf)-Gly-Asp(O^*t*^Bu)-Cys(Trt)-D-Pro-OCH_2_CO-NH-SAL-PEG resin (420 mg). A part of the resin (70 mg, 11 μmol) was treated with a TFA cocktail (TFA:TIS:H_2_O:DODT:DMB = 90:2.5:2.5:2.5:2.5, 1.5 mL) for 2 h at room temperature. The peptide was precipitated by cold diethyl ether, washed with the same solvent twice, extracted by 50% aq MeCN, and filtrated. The crude peptide was lyophilized and purified by RP-HPLC to obtain peptide **2a** (2.6 mg, 3.6 μmol, 34%). ESI mass, found m/z 707.5, calcd for (M + H)^+^: 707.3 Amino acid analysis: Asp_1.00(1.00)_Pro_0.92(1.00)_Gly_1.11(1.00)_Cys_nd__(2.00)_Arg_1.01(1.00)_.

### 4.5 H-D-Cys-Arg-Gly-Asp-Cys-D-Pro-OCH_2_CONH_2_ (2b)

To the half of Fmoc-Arg(Pbf)-Gly-Asp(OtBu)-Cys(Trt)-D-Pro-OCH_2_CO-NH-SAL-PEG Resin (61μmol) obtained during the synthesis of peptide 2a, Fmoc-D-Cys(Trt)-OH (136 mg, 0.23 mmol) was introduced to half of Fmoc-Arg(Pbf)-Gly-Asp(O^*t*^Bu)-Cys(Trt)-D-Pro-OCH_2_CO-NH-SAL-PEG resin (61 μmol) obtained during the synthesis of peptide 2a, and the Fmoc group was removed as the same manner. The resin was washed three times with MeCN and dried *in vacuo* to obtain H-D-Cys(Trt)-Arg(Pbf)-Gly-Asp(O^*t*^Bu)-Cys(Trt)-D-Pro-OCH_2_CONH-SAL-PEG resin (400 mg). A part of the obtained resin (51 mg, 7.6 μmol) was treated with a TFA cocktail (TFA:TIS:H_2_O:DODT:DMB = 90:2.5:2.5:2.5:2.5, 1.0 mL) with vortexing at room temperature for 2 h. The peptide was precipitated by adding cold diethyl ether, washing with the same solvent, and dissolving in 50% aq MeCN. After the resin was removed by filtration, the crude peptide was lyophilized, dissolved in aq MeCN, and purified by RP-HPLC to obtain peptide **2b** (1.8 mg, 2.4 μmol, 31%). ESI mass, found m/z 707.5, calcd for (M + H)^+^: 707.3; Amino acid analysis: Asp_1.00(1.00)_Pro_0.90(1.00)_Gly_1.07(1.00)_Cys_nd__(2.00)_ Arg_0.96(1.00)_.

### 4.6 H-Cys-Arg-Gly-Asp-D-Cys-Pro-OCH_2_CONH_2_ (2c)

Fmoc-NH-SAL-PEG resin (0.23 mmol/g, 650 mg, 0.15 mmol) was treated with 20% piperidine/DMF (2.0 mL) for 5 min, followed by 5 min and 10 min treatment with fresh piperidine/DMF reagents. After washing with DMF(×5), glycolic acid (24 mg, 0.31 mmol), HOBt (50 mg, 0.37 mmol), and DIC (53 μL, 0.34 mmol), DMF (2.0 mL) was added to the resin and vortexed overnight at room temperature. After washing with DMF(×5), Fmoc-Pro-OH (170 mg, 0.47 mmol), activated by 0.45 M HBTU/DMF (1.0 mL, 0.45 mmol) and DIEA (120 μL, 0.69 mmol), was added to the resin and vortexed at room temperature for 6 h. After washing with DMF (×3), followed by MeOH, the resin was dried *in vacuo*. The Fmoc group on the resin was quantified as 0.22 mmol/g (660 mg, 97%). After the Fmoc group of the resin (660 mg, 0.15 mmol) was removed and washed with DMF(×5), Fmoc-Asp(O^*t*^Bu)-D-Cys(Trt)-OH (**1b**) (230 mg, 0.31 mmol) and HOOBt (55 mg, 0.34 mmol) in DMF (2.0 mL), activated by DIC (57 μL, 0.45 mmol), were added to the resin and vortexed overnight at room temperature. The Fmoc group on the resin was quantified as 0.12 mmol/g (670 mg, 58%). The resin (74 µmol) was treated with 10% acetic anhydride and 5.0% DIEA in DMF (2.0 mL) for 5 min. After removal of the Fmoc group, Fmoc-Gly-OH, followed by Fmoc-Arg(Pbf)-OH, was introduced in the same manner described for the synthesis of peptide **2a** to obtain Fmoc-Arg(Pbf)-Gly-Asp(O^*t*^Bu)-D-Cys(Trt)-Pro-OCH_2_CONH-SAL-PEG resin. Fmoc-Cys(Trt)-OH (87 mg, 0.15 mmol) was introduced in the same manner to the half of the resin (39 μmol), followed by the Fmoc group removal to obtain H-Cys(Trt)-Arg(Pbf)-Gly-Asp(O^*t*^Bu)-D-Cys(Trt)-Pro-OCH_2_CONH-SAL-PEG resin (320 mg). A part of the resin (51 mg, 6.3 μmol) was treated with a TFA cocktail (TFA:TIS:H_2_O:DODT:DMB = 90:2.5:2.5:2.5:2.5, 1.0 mL) for 2 h at room temperature with vortexing. The peptide was precipitated by cold ether, washed with the same solvent, dissolved in 50% aq MeCN, filtrated, and lyophilized. The powder was dissolved in aq MeCN and purified by RP-HPLC to obtain peptide **2c** (1.3 mg, 1.8 μmol, 29%). ESI mass, found m/z 707.4, calcd for (M + H)^+^: 707.3. Amino acid analysis: Asp_1.00(1.00)_Pro_1.13(1.00)_Gly_1.02(1.00)_Cys_nd(2.00)_Arg_1.01(1.00)_.

### 4.7 H-D-Cys-Arg-Gly-Asp-D-Cys-Pro-OCH_2_CONH_2_ (2d)

Half of the Fmoc-Arg(Pbf)-Gly-Asp(O^*t*^Bu)-D-Cys(Trt)-Pro-OCH_2_CONH-SAL-PEG resin (38 μmol) obtained during the synthesis of peptide **2c** was treated with 20% piperidine in DMF to remove the Fmoc group, and Fmoc-D-Cys(Trt)-OH (87 mg, 0.15 mmol) was introduced as the same manner. After the removal of the Fmoc group, the resin was washed with MeCN (×3) and dried *in vacuo* to obtain H-D-Cys(Trt)-Arg(Pbf)-Gly-Asp(O^*t*^Bu)-Cys(Trt)-D-Pro-OCH_2_CONH-SAL-PEG resin (310 mg). A part of the resin (72 mg, 8.8 μmol) was treated with a TFA cocktail (TFA:TIS:H_2_O:DODT:DMB = 90:2.5:2.5:2.5:2.5, 2.0 mL) for 2 h at room temperature with vortexing. The peptide was precipitated by cold ether, washed with the same solvent, dissolved in 50% aq MeCN, filtered, and lyophilized. The powder was dissolved in aq MeCN and purified by RP-HPLC to obtain peptide **2d** (1.8 mg, 2.6 μmol, 29%). ESI mass, found m/z 707.5, calcd for (M + H)^+^: 707.3; Amino acid analysis: Asp_1.00(1.00)_Pro_0.83(1.00)_Gly_0.90(1.00)_Cys_nd(2.00)_Arg_1.02(1.00)_.

### 4.8 *cyclo*(-Cys-Arg-Gly-Asp-Cys-D-Pro-) (3a)

H-Cys-Arg-Gly-Asp-Cys-D-Pro-OCH_2_CONH_2_ **2a** (350 μg, 0.50 µmol) was dissolved in degassed 0.1 M sodium phosphate containing 20 mM TCEP・HCl and 20 mM ascorbic acid (pH 7.8, 250 µL) and allowed to react for 4 h at 37 °C under an Ar atmosphere. After 10 mM DTT (250 µL) was added, and the mixture was stirred for a few minutes, the product was isolated by RP-HPLC. The reaction yielded peptide **3a** (190 μg, 0.30 μmol, 59%). ESI mass, found m/z 632.3, calcd for (M + H)^+^: 632.2; amino acid analysis: Asp_1.00(1.00)_Pro_0.99(1.00)_Gly_1.13(1.00)_Cys_nd(2.00)_Arg_0.99(1.00)_.

### 4.9 *cyclo*(-D-Cys-Arg-Gly-Asp-Cys-D-Pro-) (3b)

H-D-Cys-Arg-Gly-Asp-Cys-D-Pro-OCH_2_CONH_2_ (**2b**) (350 μg, 0.50 µmol) was dissolved in degassed 0.1 M sodium phosphate containing 20 mM TCEP・HCl and 20 mM ascorbic acid (pH 7.8, 250 µL) and allowed to react for 4 h at 37 °C under an Ar atmosphere. After 10 mM DTT (250 µL) was added, and the mixture was stirred for a few minutes, the product was isolated by RP-HPLC. The reaction yielded peptide **3b** (190 μg, 0.27 μmol, 54%). ESI mass, found m/z 632.4, calcd for (M + H)^+^: 632.2; amino acid analysis: Asp_1.00(1.00)_ Pro_1.03(1.00)_Gly_1.06(1.00)_Cys_nd__(2.00)_Arg_0.99(1.00)_.

### 4.10 *cyclo*(-Cys-Arg-Gly-Asp-D-Cys-Pro-) (3c)

H-Cys-Arg-Gly-Asp-D-Cys-Pro-OCH_2_CONH_2_ (**2c**) (350 μg, 0.50 µmol) was dissolved in degassed 0.1 M sodium phosphate containing 20 mM TCEP・HCl and 20 mM ascorbic acid (pH 7.8, 250 µL) and allowed to react for 4 h at 37 °C under an Ar atmosphere. After 10 mM DTT (250 µL) was added, and the mixture was stirred for a few minutes, the product was isolated by RP-HPLC. The reaction yielded peptide **3c** (190 μg, 0.30 μmol, 60%). ESI mass, found m/z 632.3, calcd for (M + H)^+^: 632.2; amino acid analysis: Asp_1.00(1.00)_Pro_0.99(1.00)_Gly_1.12(1.00)_Cys_nd(2.00)_Arg_1.04(1.00)_.

### 4.11 *cyclo*(-D-Cys-Arg-Gly-Asp-D-Cys-Pro-) (3d)

H-D-Cys-Arg-Gly-Asp-D-Cys-Pro-OCH_2_CONH_2_ (**2d**) (350 μg, 0.50 µmol) was dissolved in degassed 0.1 M sodium phosphate containing 20 mM TCEP・HCl and 20 mM ascorbic acid (pH 7.8, 250 µL) and allowed to react for 4 h at 37 °C under an Ar atmosphere. After 10 mM DTT (250 µL) was added, and the mixture was stirred for a few minutes, the product was isolated by RP-HPLC. The reaction yielded peptide **3d** (180 μg, 0.25 μmol, 50%). ESI mass, found m/z 632.5, calcd for (M + H)^+^: 632.2; amino acid analysis: Asp_1.00(1.00)_ Pro_1.06(1.00)_Gly_1.29(1.00)_Cys_nd(2.00)_Arg_0.94(1.00)_.

### 4.12 *cyclo*(-Ala-Arg-Gly-Asp-Ala-D-Pro-) (4a)

*cyclo*(-Cys-Arg-Gly-Asp-Cys-D-Pro-) (**3a**) (380 μg, 0.60 µmol) was dissolved in 0.10 M citric buffer containing 0.10 M TCEP・HCl (pH 5.2, 590 µL). After 4.0 M NaBEt_4_ (15 µL) was added to the solution and allowed to react for 1 min at room temperature, the product was isolated by RP-HPLC. The reaction yielded peptide **4a** (230 μg, 0.39 µmol, 66%). ESI mass, found m/z 568.4, calcd for (M + H)^+^: 568.3; amino acid analysis: Asp_1.00(1.00)_Pro_1.01(1.00)_Gly_0.98(1.00)_Ala_2.02(2.00)_Arg_0.98(1.00)_.

### 4.13 *cyclo*(-D-Ala-Arg-Gly-Asp-Ala-D-Pro-) (4b)

*cyclo*(-D-Cys-Arg-Gly-Asp-Cys-D-Pro-) (**3b**) (370 μg, 0.60 µmol) was dissolved in 0.10 M citric buffer containing 0.10 M TCEP・HCl (pH 5.2, 590 µL). After 4.0 M NaBEt_4_ (15 µL) was added to the solution and reacted for 1 min at room temperature, the product was isolated by RP-HPLC. The reaction yielded peptide **4b** (230 μg, 0.39 µmol, 67%). ESI mass, found m/z 568.5, calcd for (M + H)^+^: 568.3; amino acid analysis: Asp_1.00(1.00)_Pro_1.00(1.00)_Gly_0.98(1.00)_Ala_2.02(2.00)_Arg_0.99(1.00)_.

### 4.14 *cyclo*(-Ala-Arg-Gly-Asp-D-Ala-Pro-) (4c)

*cyclo*(-Cys-Arg-Gly-Asp-D-Cys-Pro-) (**3c**) was dissolved in 0.10 M citric buffer containing 0.10 M TCEP・HCl (pH 5.2, 590 µL). After 4.0 M NaBEt_4_ (15 µL) was added to the solution and allowed to react for 1 min at room temperature, the product was isolated by RP-HPLC. The reaction yielded peptide **4c** (270 μg, 0.48 µmol, 76%). ESI mass, found m/z 568.5, calcd for (M + H)^+^: 568.3; Amino acid analysis: Asp_1.00(1.00)_Pro_1.03(1.00)_ Gly_0.99(1.00)_Ala_2.02(2.00)_ Arg_1.04(1.00)_.

### 4.15 *cyclo*(-D-Ala-Arg-Gly-Asp-D-Ala-Pro-) (4d)

*cyclo*(-D-Cys-Arg-Gly-Asp-D-Cys-Pro-) (**3d**) was dissolved in 0.10 M citric buffer containing 0.10 M TCEP・HCl (pH 5.2, 590 µL). After 4.0 M NaBEt_4_ (15 µL) was added to the solution and allowed to react for 1 min at room temperature, the product was isolated by RP-HPLC. The reaction yielded peptide **4d** (230 μg, 0.39 µmol, 73%). ESI mass, found m/z 568.4, calcd for (M + H)^+^: 568.3; amino acid analysis: Asp_1.00(1.00)_Pro_1.07(1.00)_Gly_1.02(1.00)_ Ala_1.99(2.00)_Arg_0.97(1.00)_.

### 4.16 *cyclo*(-Cys(Me)-Arg-Gly-Asp-Cys(Me)-D-Pro-) (5a)

H-Cys-Arg-Gly-Asp-Cys-D-Pro-OCH_2_CONH_2_ (**2a**) (350 μg, 0.50 µmol) was dissolved in degassed 0.1 M sodium phosphate containing 20 mM TCEP・HCl and 20 mM ascorbic acid (pH 7.8, 250 µL), and the mixture was vortexed at 37 °C for 4 h under an Ar atmosphere. DMF (25 μL). Then 1.0 M CH_3_I in DMF (25 μL, 25 μmol) was added, and the solution was vortexed at 37 °C for 30 min under an Ar atmosphere. After 1.0 M DTT/DMF (30 μL, 30 μmol) was added to terminate the reaction, the product was purified by RP-HPLC to obtain peptide **5a** (160 μg, 0.24 µmol, 48%). ESI mass, found m/z 660.5, calcd for (M + H)^+^: 660.3; amino acid analysis: Asp_1.00(1.00)_Pro_0.92(1.00)_Gly_1.09(1.00)_ Cys_nd(2.00)_Arg_1.00(1.00)_.

### 4.17 *cyclo*(-D-Cys(Me)-Arg-Gly-Asp-Cys(Me)-D-Pro-) (5b)

H-D-Cys-Arg-Gly-Asp-Cys-D-Pro-OCH_2_CONH_2_ (**2b**) (350 μg, 0.50 µmol) was dissolved in degassed 0.1 M sodium phosphate containing 20 mM TCEP・HCl and 20 mM ascorbic acid (pH 7.8, 250 µL), and the mixture was vortexed at 37 °C for 4 h under an Ar atmosphere. DMF (25 μL). Then, 1.0 M CH_3_I in DMF (25 μL, 25 μmol) was added, and the solution was vortexed at 37 °C for 30 min under an Ar atmosphere. After 1.0 M DTT/DMF (30 μL, 30 μmol) was added to terminate the reaction, the product was purified by RP-HPLC to obtain peptide **5b** (150 μg, 0.23 µmol, 45%). ESI mass, found m/z 660.5, calcd for (M + H)^+^: 660.3; amino acid analysis: Asp_1.00(1.00)_Pro_0.90(1.00)_ Gly_1.07(1.00)_Cys_nd(2.00)_Arg_0.99(1.00)_.

### 4.18 *cyclo*(-Cys(Me)-Arg-Gly-Asp-D-Cys(Me)-Pro-) (5c)

H-Cys-Arg-Gly-Asp-D-Cys-Pro-OCH_2_CONH_2_ (**2c**) (350 μg, 0.50 µmol) was dissolved in degassed 0.1 M sodium phosphate containing 20 mM TCEP・HCl and 20 mM ascorbic acid (pH 7.8, 250 µL), and the mixture was vortexed at 37 °C for 4 h under an Ar atmosphere. DMF (25 μL). Then, 1.0 M CH_3_I in DMF (25 μL, 25 μmol) was added, and the solution was vortexed at 37 °C for 30 min under an Ar atmosphere. After 1.0 M DTT/DMF (30 μL, 30 μmol) was added to terminate the reaction, the product was purified by RP-HPLC to obtain peptide **5c** (160 μg, 0.24 µmol, 47%). ESI mass, found m/z 660.5, calcd for (M + H)^+^: 660.3; amino acid analysis: Asp_1.00(1.00)_Pro_0.92(1.00)_ Gly_1.08(1.00)_ Cys_nd(2.00)_Arg_1.00(1.00)_.

### 4.19 *cyclo*(-D-Cys(Me)-Arg-Gly-Asp-D-Cys(Me)-Pro-) (5d)

H-D-Cys-Arg-Gly-Asp-D-Cys-Pro-OCH_2_CONH_2_ (**2d**) (350 μg, 0.50 µmol) was dissolved in degassed 0.1 M sodium phosphate containing 20 mM TCEP・HCl and 20 mM ascorbic acid (pH 7.8, 250 µL), and the mixture was vortexed at 37 °C for 4 h under an Ar atmosphere. DMF (25 μL). Then, 1.0 M CH_3_I in DMF (25 μL, 25 μmol) was added, and the solution was vortexed at 37 °C for 30 min under an Ar atmosphere. After 1.0 M DTT/DMF (30 μL, 30 μmol) was added to terminate the reaction, the product was purified by RP-HPLC to obtain peptide **5d** (150 μg, 0.23 µmol, 45%). ESI mass, found m/z 660.5, calcd for (M + H)^+^: 660.3; amino acid analysis: Asp_1.00(1.00)_Pro_0.88(1.00)_ Gly_1.01(1.00)_Cys_nd(2.00)_Arg_1.00(1.00)_.

### 4.20 Integrin inhibition assay with synthetic peptides

The integrin inhibition assay was performed as follows. The 96-well Nunc MaxiSorp™ flat-bottom plate was coated overnight with 50 µL of a 10 nM LAP solution at 4°C. After washing with 200 µL of Tris-buffered saline [20 mM Tris-HCl (pH 7.4) containing 137 mM NaCl] (TBS) containing 0.1% (v/v) Tween-20 (Sigma) and 3% (w/v) BSA (Sigma) (3% BSA/TBS-T), the plate was blocked with 200 µL of 3% BSA/TBS-T for 1 h at room temperature. After washing three times with TBS containing 0.1%(v/v) Tween-20 (Sigma), 0.3% (w/v) BSA (Sigma), and 1 mM manganese chloride (Wako) (Mn-Wash), 50 µL of 10 nM αvβ6 integrin preincubated with 1 µM peptide in Mn-Wash for 1 h at room temperature was added to the wells and incubated with gentle agitation for 1 h at room temperature. The bound αvβ6 integrin was detected by the biotinylated anti-Velcro polyclonal antibody and horseradish peroxidase-conjugated streptavidin. In brief, after washing three times with the Mn-Wash, the plate was incubated with 50 µL of 1.5 μg/mL biotinylated anti-Velcro antibody for 0.5 h at room temperature with gentle agitation. The bound antibody was detected by horseradish peroxidase-conjugated streptavidin (0.53 μg/mL), followed by colorimetrically measuring the amount of bound αvβ6 integrin using *o*-phenylenediamine (0.4 mg/mL; FUJIFILM Wako) and H_2_O_2_ (0.006%) in a 25 mM citric acid/50 mM Na_2_HPO_4_ buffer. The colorimetric reaction was stopped with 100 μL of 2.5 M H_2_SO_4_, and the absorbance of the chromogenic substrate was measured at 490 nm using a microplate reader (Molecular Devices EMax). A_490_-A_650_ was used to calculate the inhibitory activity. The inhibition activities of the synthetic peptides for the αvβ6 integrin were calculated according to the following formula:

$$Inhibitory\;activity\;\left(\%\right)=100-\frac{Ap-Ab}{Ai-Ab}\times 100,$$

where *A*_*p*_ is the absorbance at 490 nm of the bound αvβ6 integrin with synthetic peptide, *A*_*i*_ is the absorbance at 490 nm of the bound αvβ6 integrin without synthetic peptide (control), and *A*_*b*_ is the absorbance at 490 nm of 1 mM manganese chloride as a background.

The inhibitory activity was calculated as the mean of three wells per analysis. All the measurements were performed with N = 3, and the final inhibitory activity was calculated as the mean ± standard error.

## Data Availability

The original contributions presented in the study are included in the article/Supplementary Material; further inquiries can be directed to the corresponding author.
