# Supplementary material for: Application of cysteinyl prolyl ester for the synthesis of cyclic peptides containing an RGD sequence and their biological activity measurement
Source: Front Chem. 2024 May 30;12:1391678. doi: 10.3389/fchem.2024.1391678 (PMC11169864; doi:10.3389/fchem.2024.1391678)
Supplement: Supplementary file 1 [file DataSheet1.PDF]

Supporting information

Application of cysteinyl prolyl ester for the synthesis of cyclic peptides containing RGD sequence and their biological activity measurement

Akina Yamada, Toshiki Takei, Toru Kawakami, Yukimasa Taniguchi, Kiyotoshi Sekiguchi, Hironobu Hojo*

Institute for Protein Research, Osaka University, Suita, Osaka 565-0871, Japan

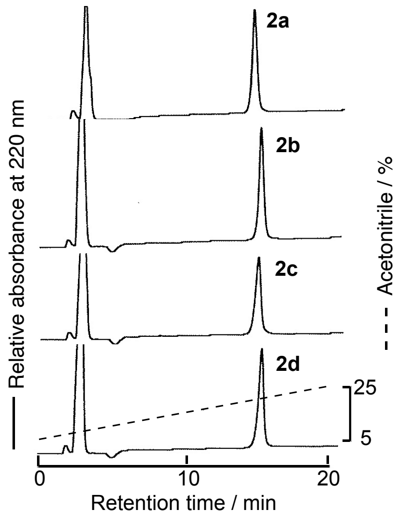

Column: YMC-Triart C18 150×4.6 mm
Eluent: MeCN/H_2O containing 0.10% TFA
Flow rate: 1.0 mL/min

Fig. SI-1. RP-HPLC profile of purified peptides **2a-2d**.

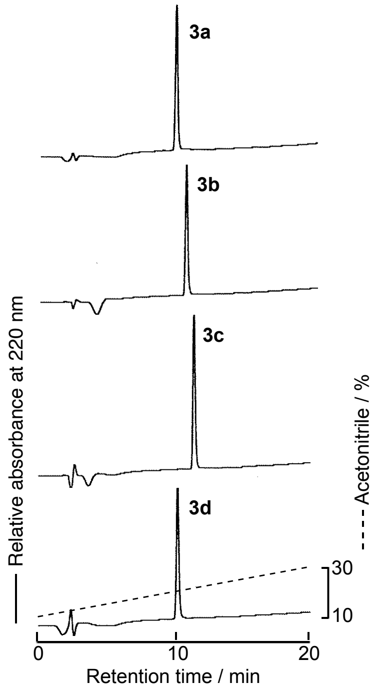

Column: YMC-Triart C18 150×4.6 mm
Eluent: MeCN/H_2O containing 0.10% TFA
Flow rate: 1.0 mL/min

Fig. SI-2. RP-HPLC profile of purified peptides **3a-3d**.

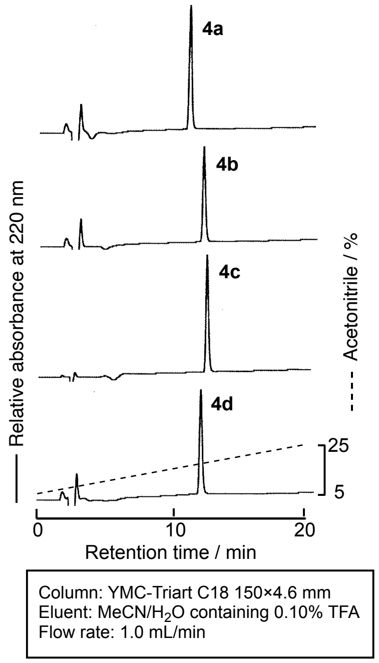

Fig. SI-3. RP-HPLC profile of purified peptides **4a**-**4d**.

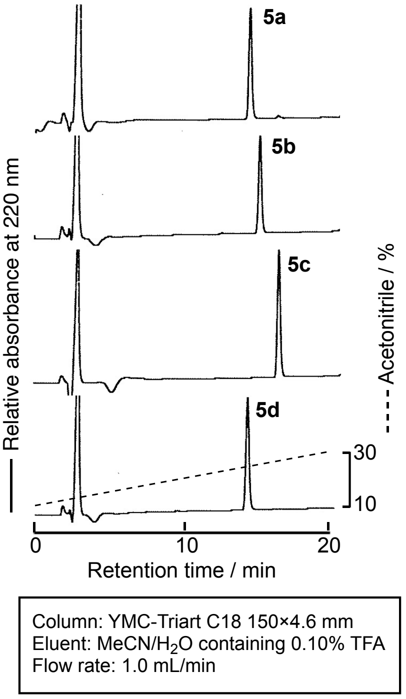

Fig. SI-4. RP-HPLC profile of purified peptides **5a**-**5d**.
